# Investigation on compression and mildew of mixed and separated maize

**DOI:** 10.1002/fsn3.2985

**Published:** 2022-07-27

**Authors:** Chaosai Liu, Guixiang Chen, Yang Zhou, Longfei Yue, Wenlei Liu

**Affiliations:** ^1^ College of Civil Engineering Henan University of Technology Zhengzhou China; ^2^ Henan International Joint Laboratory of Modern Green Ecological Storage System Zhengzhou China; ^3^ Henan Key Laboratory of Grain Storage Facility and Safety Zhengzhou China

**Keywords:** creep behavior, heat production, Kernel distribution, maize, vertical pressure

## Abstract

This study explores the influence of different segregation configurations on the creep behaviors and mildew of maize. An inexpensive and easy‐to‐use system was designed, and three configurations of maize kernels distribution, i.e., uniform mixing (Md_m_), alternating distribution (Md_a_), and segregated state distribution (Md_s_), with wet basis moisture content of 22.9%, were compressed under vertical pressure of 200 kPa through a one‐dimensional oedometer. The compression and creep behaviors were investigated using the strain/settlement*–*time results, and aerobic plate counting (APC) was performed to study the effect of distribution configuration on the mildew effect. A finite‐element model was established to simulate the temperature variation caused by physical environmental factors, and the heat production by fungi was quantified using the difference in temperature between simulation and test. The results indicate that the three‐element Schiffman model can represent the creep behavior of the maize with different distribution configurations. The average temperature of Md_m_, Md_a_, and Md_s_ were 7.53%, 12.98%, and 14.76% higher than the average room temperature, respectively. The aerobic plate count of Md_m_, Md_a_, and Md_s_ were 1.0 × 10^5^, 2.2 × 10^5,^ and 8.8 × 10^5^ cfu g^−1^ stored for 150 h, respectively. In general, the temperature and APC in segregated maize bulk are higher than uniform grain. The effectiveness of the numerical model was verified, and the heat production by maize bulk fungi was quantified using the test and numerical temperature difference. The average heat was the least in Md_m_ with 2.8 × 10^6^ J m^−3^, and Md_a_ and Md_s_ were 1.7 and 2 times more than Md_m_. And the heat was related to the segregation configurations and agreed very well with the APC and temperature results.

## Graphical abstract

This study explores the influence of three segregation configurations on the creep behaviors and mildew of maize. A newly empirical analytical‐numerical method was established to evaluate the heat generated by fungi, considering segregation configuration and compression. The aerobic plate count (APC) and the temperature of in segregated maize bulk are higher than uniform grain. And the heat production was related to the segregation configurations and agreed very well with the APC and temperature results.

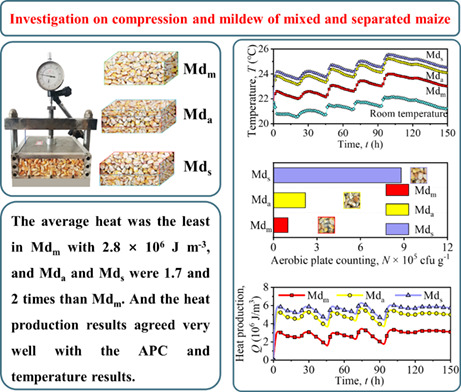

## 1 INTRODUCTION

The acreage and annual production output of maize in China are over 40 million hectares and 260 million tons, accounting for 35.3% and 38.9% of the total grain acreage and output, respectively (National Bureau of Statistics, 2020), and it plays an important role in increasing grain production and farmers' income. COVID‐19 outbreak has drastically aggravated the stresses on the food systems (FAO, 2020a, 2020b; Goswami et al., 2021). Safe and economical storage of such a larger amount of maize is significantly important.

Maize kernel breakage and segregation is one of the important problems in harvesting and postharvest processing (Shahbazi et al., 2017). When a bulk of maize containing lots of broken kernels are particularly prone to segregate whenever they are processed, e.g., forming a heap in silos during top filling (Nourmohamadi‐Moghadami, Zare, Singh, & Stroshine, 2020; Nourmohamadi‐Moghadami, Zare, Stroshine, & Kamfiroozi, 2020). The whole and broken kernels are different in some properties such as size, shape, density, and particle surface roughness. Different distribution configurations may appear under the different segregation actions (i.e., trajectory, fluidization, shifting, and impact) when maize kernels drop and flow on the surface of the grain heap (Jian et al., 2019). Fan et al. (2017) divided the configurations into three categories: (1) large and small kernels remain mixed without segregation; (2) large and small kernels form alternating layers, resulting in a stratified state distribution; and (3) large and small kernels form a segregated state distribution. Another issue is that grain mildew was affected by the factors. Many research have focused on the effect of temperature and moisture content (Adams & Rosentrater, 2021; Christensen & Kaufmann, 1965; Quemada‐Villagomez et al., 2020) on grain mildew. However, some scholars argued that kernel breakage (Mohapatra et al., 2017), segregation (Jian et al., 2019), and compression (Mohapatra et al., 2017; Su et al., 2019) also impact grain mildew significantly, however, more related investigations are still lacking.

First, coupled heat and moisture transfer in grain bulk has been studied extensively and valuable results have been obtained such as natural convection by Khankari et al. (1994) and Wang et al. (2016), and the ventilation drying process by Thorpe (2008) and Panigrahi et al. (2019). Furthermore, some researchers confirmed that segregation led to non‐uniform airflow distribution, which further resulted in higher temperature and higher relative humidity in zones with low airflow in the same silo (Navarro & Noyes, 2001; Yue & Zhang, 2017). Most researchers are devoted to the study of heat and moisture transfer in grain bulk (Hammami et al., 2016; Wang et al., 2016) and the relationship between segregation and airflow resistance (Lawrence & Maier, 2011). Moreover, the grain bulk will unevenly creep with the storage period at a given storage height, and the creep behavior will significantly influence the maize quality during and after storage (Moreira et al., 2015).

However, the above research have neither considered the combined effect of kernel breakage and its induced segregation on mildew nor there is information in the literature about the relationship between the compression of broken maize kernels due to vertical loading and mildew. On one hand, breakage played a major role in the density distribution (Navarro & Noyes, 2001) and the redistributed density further affects the thermal conductivity. It was well known that with increasing bulk density, the thermal conductivity increased accordingly because the contact area between kernels influences heat transfer (Chang, 1986; Cheng et al., 2017), resulting in the redistribution of moisture content and heat in the grain bulk. The redistribution of moisture content and heat may initiate the growth of massive molds that start growing in the grain domain and the release of large amount of heat (Jian et al., 2019), resulting in maize deterioration, reduction of grain mass, liberation of flavors, and existence of several mycotoxins (Olstorpe et al., 2010; Suleiman et al., 2018; Burger et al., 2013). And on the other hand, the broken maize kernels were more likely to be contaminated and invaded by fungi compared with the whole kernels (Mohapatra et al., 2017). Up to date, there are rare research on the mildew induced by kernel breakage and its induced segregation.

Grain mildew is mainly manifested in heat production, so quantitative heating is of great significance to the study of mildew. Wu et al. (2020) proposed the solution for heat production in wheat grain bulk, considering fungal activities induced temperature rise, it laid a foundation for the calculation of heat production by fungi. However, this method is not easy to perform as it needs to be calculated through the comparison of temperature before and after grain mildew with long‐term storage, and the density change in maize bulk with different distribution configurations caused by vertical loading was not considered. Therefore, the objective of this study was to investigate the effects of three representative maize kernels distribution configurations on mildew under the same vertical pressure. The creep behavior of maize bulk was analyzed, and the density at different times was obtained. Then, the heat and moisture transfer computational model of maize bulk was established and validated by the experiment. Subsequently, the temperature change of maize bulk without mildew at room temperature was numerically studied and compared with that of mildewed maize bulk in this environment. Finally, the heat generated by fungi was quantified according to experimental and numerical data, which is of great significance to guide the calculation of heat production of fungi in grain bulk.

## 2 MATERIALS AND METHODS

### 2.1 Equipment

**FIGURE 1:**
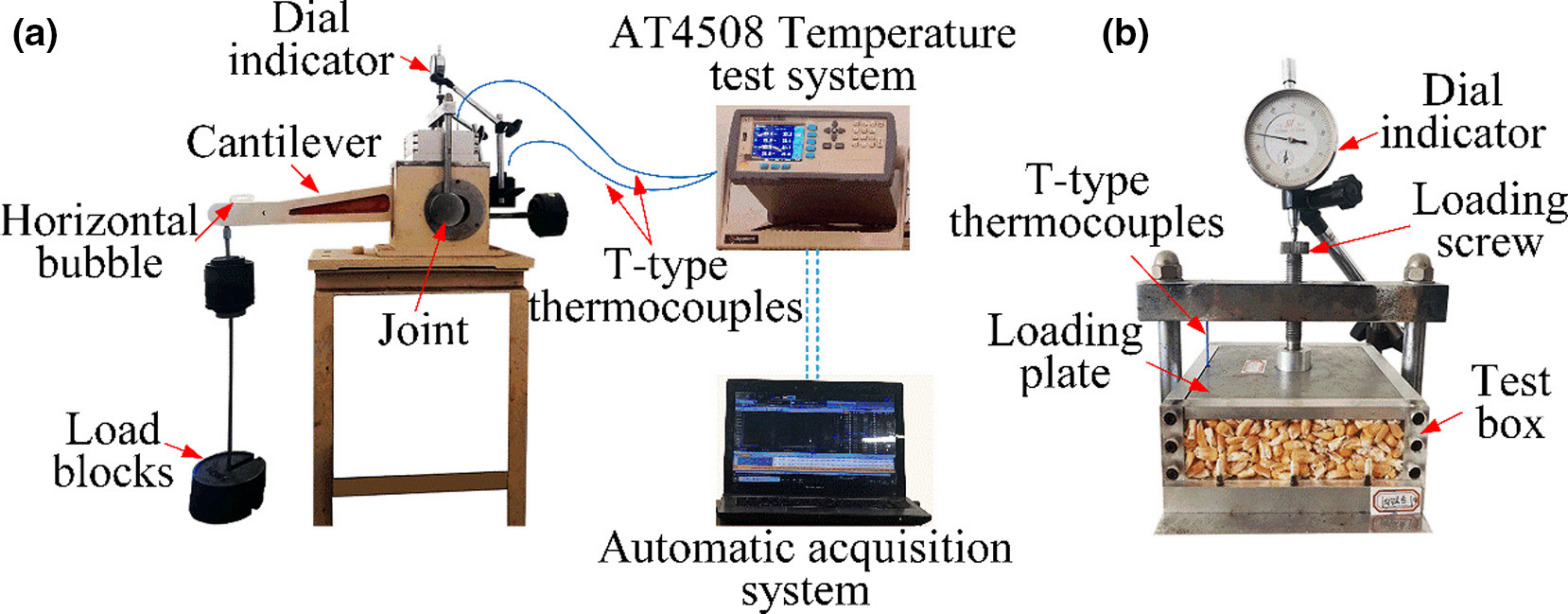
Test system, (a) schematic diagram and (b) sample cell

The apparatus used in this study (Figure 1) was modified from a conventional oedometer, normally used for soil testing. The apparatus consists of a loading frame, a box, and a measuring system. The loading was applied from the top plate, above the cell through a lever and dead weight. The box is made of high‐strength aluminum alloy and plexiglass with a height of 55 mm, with an internal size of 120 × 120 mm. The metal loading plate is highly stiff with a flexible rubber pad pasted below it (Talesnick et al., 2008). The vertical displacement (*s*) and temperatures (*T*) were measured during the test by a dial indicator and two T‐type thermocouples (Applent Instruments Inc.), respectively. The dial indicator was set on the loading screw. One thermocouple was placed in the geometric center of the maize sample and another was attached to the external side of the box to observe the room temperature. The temperature results were recorded with an AT4508 temperature testing system (Applent Instruments Inc.) and an automatic acquisition system.

### 2.2 Test material

**FIGURE 2:**
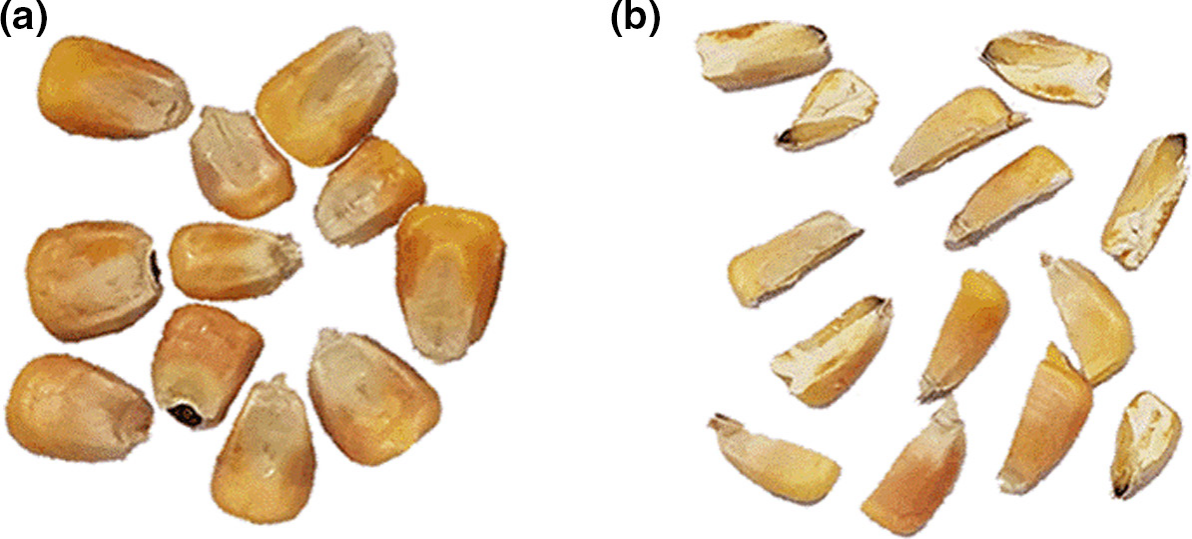
Maize kernels, (a) whole maize kernels (WK) and (b) half kernels (HK) cut along the middle line

The maize used in this experiment was Zhengdan 958 hybrid maize, harvested in 2020. The harvested maize was filtered through a circular sieve to remove the broken grains and foreign impurities. A total of three maize samples were randomly selected from the filtered grains. Half of the maize kernels were cut into two parts along the middle line of maize kernel, as shown in Figure 2b. According to ASAE (2017a), the moisture content of maize can be measured after drying at 103°C for 72 h. Finally, the moisture content of the original maize was determined to be 10.3 ± 0.02%. In this study, we focused on the numerical simulation and analytical investigation of the mildew process. Thus, we increased the moisture content to about 23% to shorten the test duration which might not be the optimum moisture content for storage in reality. To reach the target moisture content, we sprayed distilled water and mixed it with the sample thoroughly. Then, the samples were hermetically sealed in polyethylene bags and stored at 4°C for 48 h to allow moisture equilibrium (Suleiman et al., 2018). The actual moisture content of maize after reaching moisture equilibrium was 22.9 ± 0.06%. The mean of standard maize bulk density (*ρ*_*b*0_) of Md_m_, Md_a,_ and Md_s_ were 670.2 ± 0.7, 671.9 ± 0.4, and 641.6 ± 0.6 kg m^−3^, respectively.

**FIGURE 3:**
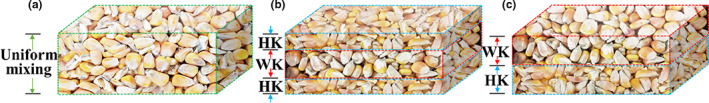
Three configurations of maize kernel distribution, (a) uniform mixing (Md_m_), (b) alternating distribution (Md_a_), and (c) segregated state distribution with whole kernels at the upper and half kernels at the lower (Md_s_)

First, the walls of the testing box were coated with a thin layer of Vaseline to eliminate side friction, and then a 400 g sample of maize kernels was randomly taken and deposited into the testing box in three ways: (1) Uniform mixing of 200 g whole kernels and 200 g half kernels (Md_m_), and this form was called low degree of separation; (2) Whole and half kernels form alternating layers (Md_a_), 100 g half kernels were first deposited into the test box, then 200 g whole kernels were deposited, and the other 100 g half kernels were finally deposited. This form was called a high degree of separation. (3) Whole and half kernels form a segregated state distribution (Md_s_). 200 g half kernels were first deposited into the test box, and then 200 g whole kernels were deposited. This form was called complete separation with a higher degree of separation (Figure 3).

### 2.3 Test procedure

#### 2.3.1 Loading

As the diameter of the loading plate in the conventional oedometer test is 61.8 mm, it was different from the sample area in this study; the stress applied to the maize should be estimated prior. After calculation, the vertical stress applied followed a sequence of 3, 13, 34, 76, 117, 159, and 200 kPa. In this study, three tests (i.e., Md_m_, Md_a_, and Md_s_) were performed, and load block was applied to 200 kPa by multi‐stages. The cantilever drove the loading screw to move downward, and the corresponding load was applied, as shown in Figure 1. It is worth noting that each loading stage lasted for 10 min before reaching the final stress. Then, the three tests were continued for 150 h at constant stress. After the loading test, the APC test was performed to check the mildew and grain quality.

#### 2.3.2 Aerobic plate counting

**FIGURE 4:**
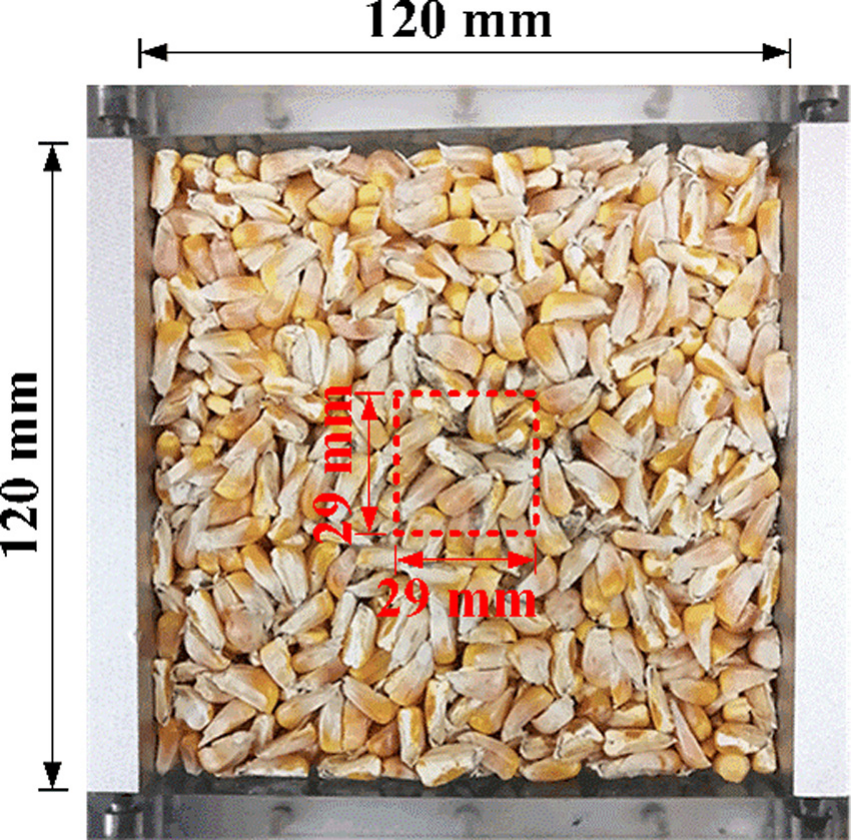
Sampling points for aerobic plate counting (APC)

Maize kernels at the center of the box, as shown in Figure 4, were selected to conduct the APC test. The sample was prepared using a rectangular plastic sampler with a size of 29 × 29 mm, which was used to take out about 30 g of grain.

The specific preparation process for the APC test is as follows:

1. Preparation of the plate counting agar (PCA). First, the tryptone, yeast extract, glucose, and agar were mixed thoroughly and dissolved fully, and the pH was controlled at 7.0 ± 0.2. The mixed solution was autoclaved at 121°C for 15 min in a conical flask.
2. Preparation of the bacterial suspension. Twenty‐five grams of the maize sample and 225 ml of sterile water (National Standards of the People's Republic of China, 2016) were put into a 500 ml conical flask together. The conical flask was placed in an oscillator and vibrated for 30 min. The aim of this process was to fully diffuse the microorganisms on the surface of the maize kernels into the sterile water. By this process, a homogenate with a concentration of 1:10 was obtained.
3. Preparation of homogenates with different concentrations by serial dilution. One milliliter of 1:10 sample homogenate was drawn by a 1000 μl pipette, and was mixed in a test tube containing 9 ml sterile water to obtain a sample homogenate with 1:100 concentration. The above steps were repeated several times to obtain a series of solutions with different concentrations. Normally, two homogenates with different concentrations should be prepared for later fungus culture depending on the contamination conditions. In this study, sample homogenates with concentrations of 1:10 and 1:100 were selected for maize before testing, and 10^−5^ and 10^−6^ were selected after the test, for comparison.
4. *Fungus culture*: One milliliter of sample homogenate with desired concentration was taken and mixed with 15 ~ 20 ml PCA. Then, the mixture was cooled to 46°C before injecting into three presterilized Petri dishes. After the agar was coagulated, the dishes were turned over and cultured at 30 ± 1°C for 72 h (MJPS‐150, Shanghai Jing Hong Laboratory Instrument Co.).
5. *Colony counting*: For each sample, select one from the three dishes with mold colony number ranging from 100 ~ 150 colony‐forming units (cfus) and the type and number of colonies were recorded by observing under a microscope. The number of colonies was calculated as follows:

(1) $$N=\frac{\Sigma C}{\left(n_{1}+0.1n_{2}\right)\;d}$$

**FIGURE 5:**
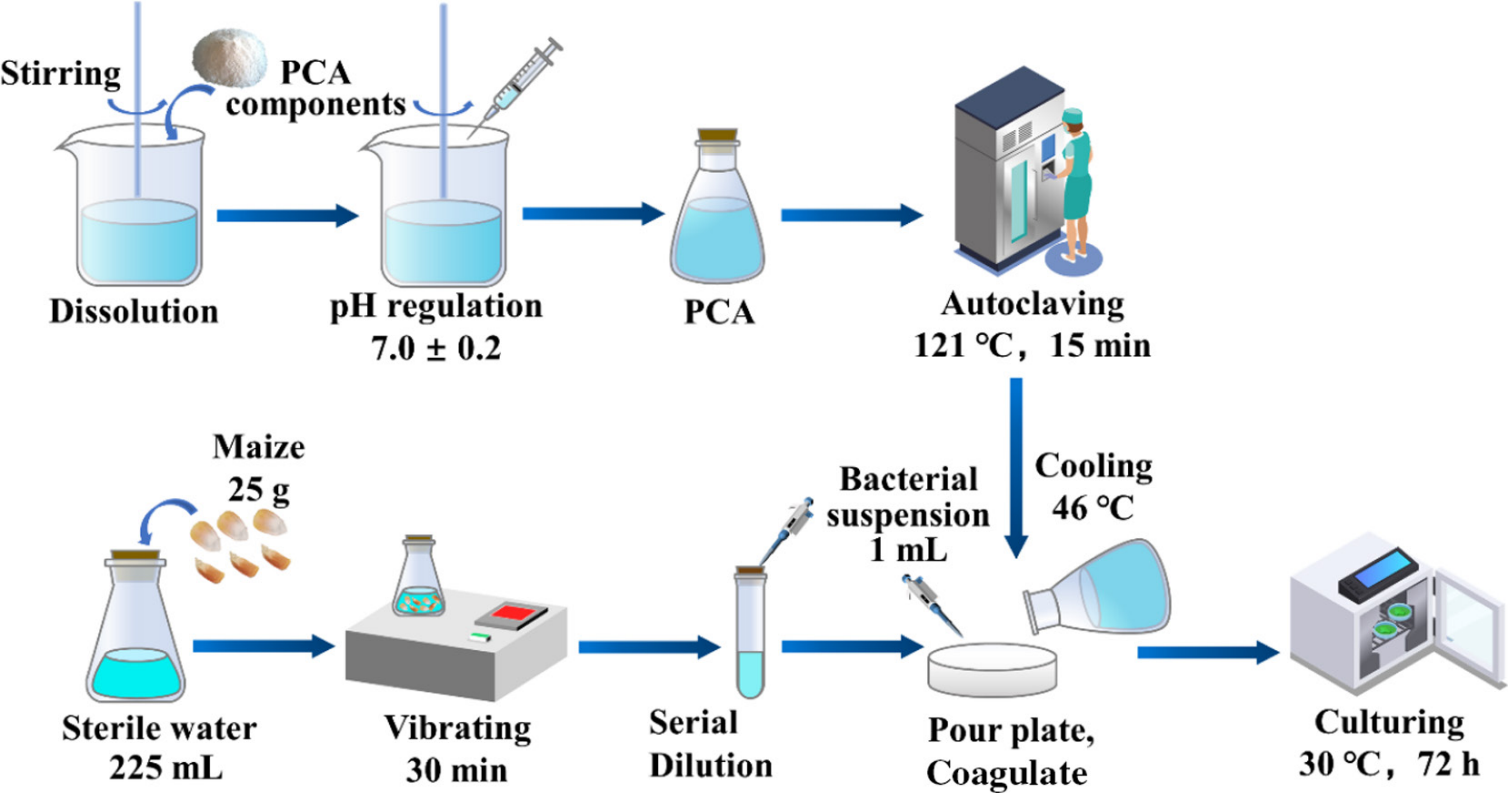
Process of the aerobic plate counting (APC) test

Where *N* is the aerobic plate count; ΣC is the sum of bacterial colony number; $n_{1}$ is the number of colonies for 1:10 homogenate; $n_{2}$ is the number of colonies for 1:100 homogenate; and *d* is the dilution. In this study, *d* is equal to 10^−1^ for maize before the test, and 10^−5^ after the test. The procedure for the APC test is shown in Figure 5. The initial APC is 1.7 × 10^2^ cfu g^−1^.

## 3 RESULTS

### 3.1 Creep behavior of maize bulk

**FIGURE 6:**
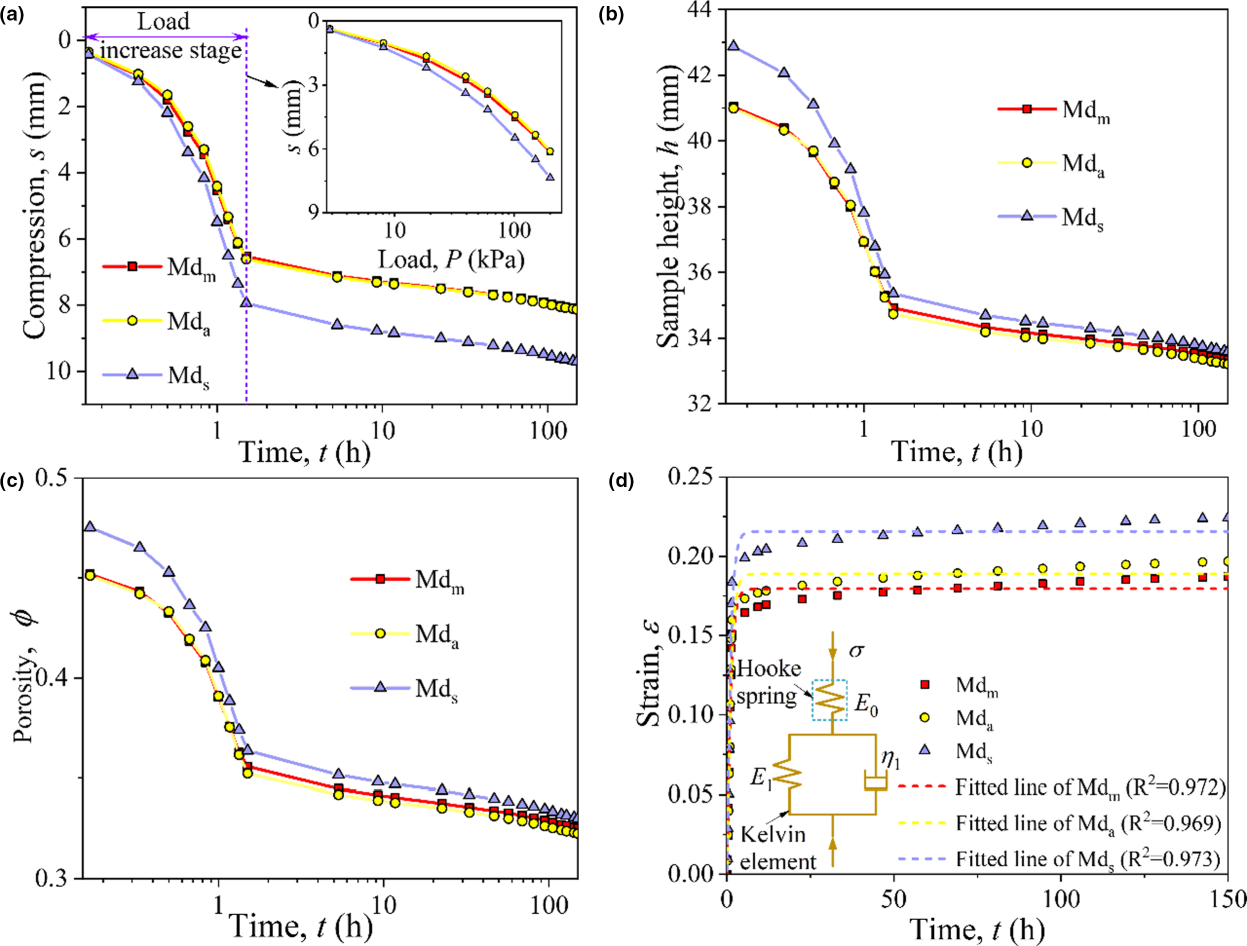
Deformation of maize under vertical load, (a) compression deformation, (b) sample height, (c) porosity, and (d) creep model

Creep deformation analysis (i.e., creep) is considered a valuable method in the quality assessment of grain/food products (Moreira et al., 2015). The deformation of maize under vertical load (i.e., gravity or weight of machine) results in a reduction in void ratio and ultimately impacts the moisture content, temperature, and biochemical functions. The acidity and protein quality will also be significantly influenced by considerable compression (Hadnađev et al., 2015; Sheng et al., 2014). The density difference formed by grain segregation results in uneven sedimentation (Cheng et al., 2017). The compression (*s*)*–*time (*t*) curve on semilog coordinates is shown in Figure 6a. The segregation state and initial density impact the vertical compression of maize. The compression increased with the increase in the degree of separation. The compression of Md_m_, Md_a_, and Md_s_ were 8.1, 8.14, and 9.7 mm, respectively, under 200 kPa (Figure 6a). The deformation in the stage of load increase was the main cause of compression deformation, which accounted for 80.6%, 81.3%, and 81.8% of the total deformation of Md_m_, Md_a_, and Md_s_ respectively. The corresponding sample height is seen in Figure 6b. The compression deformation of Md_s_ sample is large at the load increase stage due to the difference in initial density. The porosity decreased by 22.2%, 22.7%, and 24.3%, respectively, in load increase stage, and it tended to be consistent with time (Figure 6c). The *s –* log (*t*) curve follows a reversed *s*‐shape, and the inflection point indicates the end of elastic deformation and the beginning of creep. It can be seen clearly that the sample soon entered the creep stage without any incremental load, i.e., after the final loading.

The strain (*ε*) ~ *t* relationship of granular materials (e.g., sand) is normally analyzed by the Schiffman model (Sheng et al., 2014) with a Maxwell element and a Kelvin element, as seen in Figure 6d. The Schiffman model can be expressed by Equation (2):

(2) $$\varepsilon =\frac{\sigma }{E_{0}}+\frac{\sigma }{E_{1}}\left(1-e^{-\frac{E_{1}}{\eta_{1}}}\right)+\frac{\sigma }{\eta }t$$

Where *ε* is the total strain at time *t*, σ is the applied stress, $E_{0}$ is the elastic modulus, $E_{1}$ is the deformation modulus of creep stage, $\eta_{1}$ is the viscosity coefficient of Kelvin element, and η is the viscosity coefficient of Maxwell element.

All regression parameters have the coefficient of determination *R* ^2^ > .95, which observation suggests that the three‐element Schiffman model can represent the creep behavior of different distribution configurations. The deformation mainly underwent three stages. (1) Instantaneous deformation (0–1.5 h): the deformation was mainly affected by the magnitude of the load. The compression deformation occurred almost at the moment of load application, and the kernels slipped along the contact surface due to shear stress overcoming the friction at the contact surface. The broken kernels fill the large pores between kernels. (2) Rapid deformation (1.5–24 h): the curve in this stage is mainly a section near the inflection point, the kernels were compressed, the pores between the kernels were filled, the resistance of the kernels increases, and the deformation rate decreases. (3) Steady‐state creep (24–150 h): the pores between kernels were basically filled, the resistance of kernels was greater, and the creep curve tended to be flat, roughly parallel to the abscissa axis. The creep behavior of maize bulk is greatly affected by relative density.

### 3.2 Temperature change

**FIGURE 7:**
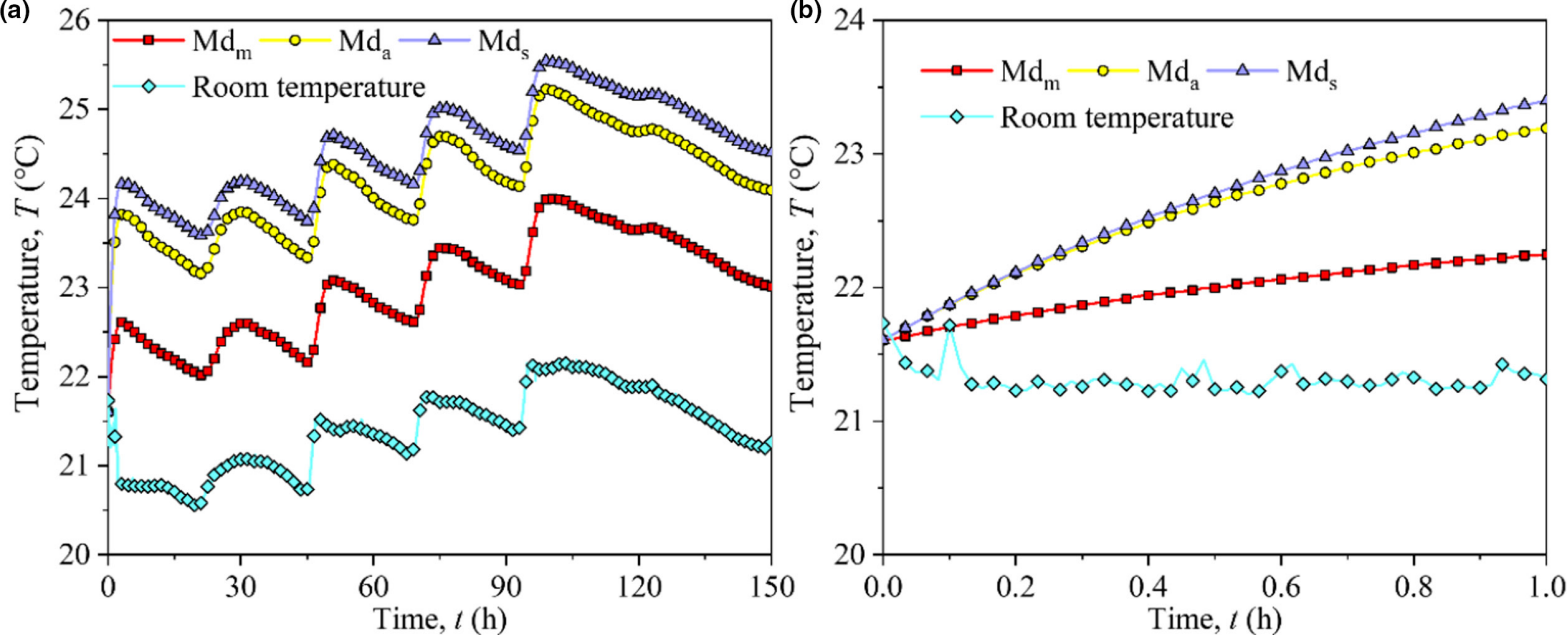
Temperature change of three configurations of maize kernels distribution, (a) storage for 150 h and (b) storage for 1 h

Due to respiration in wet maize kernels and microorganisms, a considerable amount of heat is released, thus raising the grain temperature to rise; therefore, the temperature difference is a sensitive indicator of maize mold activity (Mohapatra et al., 2017). The temperature of maize bulk with different distribution configurations is shown in Figure 7. By observing the room temperature, we found that heating intensity was high during daytime and low during nighttime. The temperature of maize bulk was significantly influenced by the laboratory environment and was higher than the room temperature. There were considerable differences among the different distribution configurations. The temperature was the highest in Md_s_ and lowest in Md_m_. The average temperatures of Md_m_, Md_a_, and Md_s_ were 23.01, 24.18, and 24.56°C, respectively, which are 7.53%, 12.98%, and 14.76% higher than the average room temperature, respectively.

### 3.3 Aerobic plate count

**FIGURE 8:**
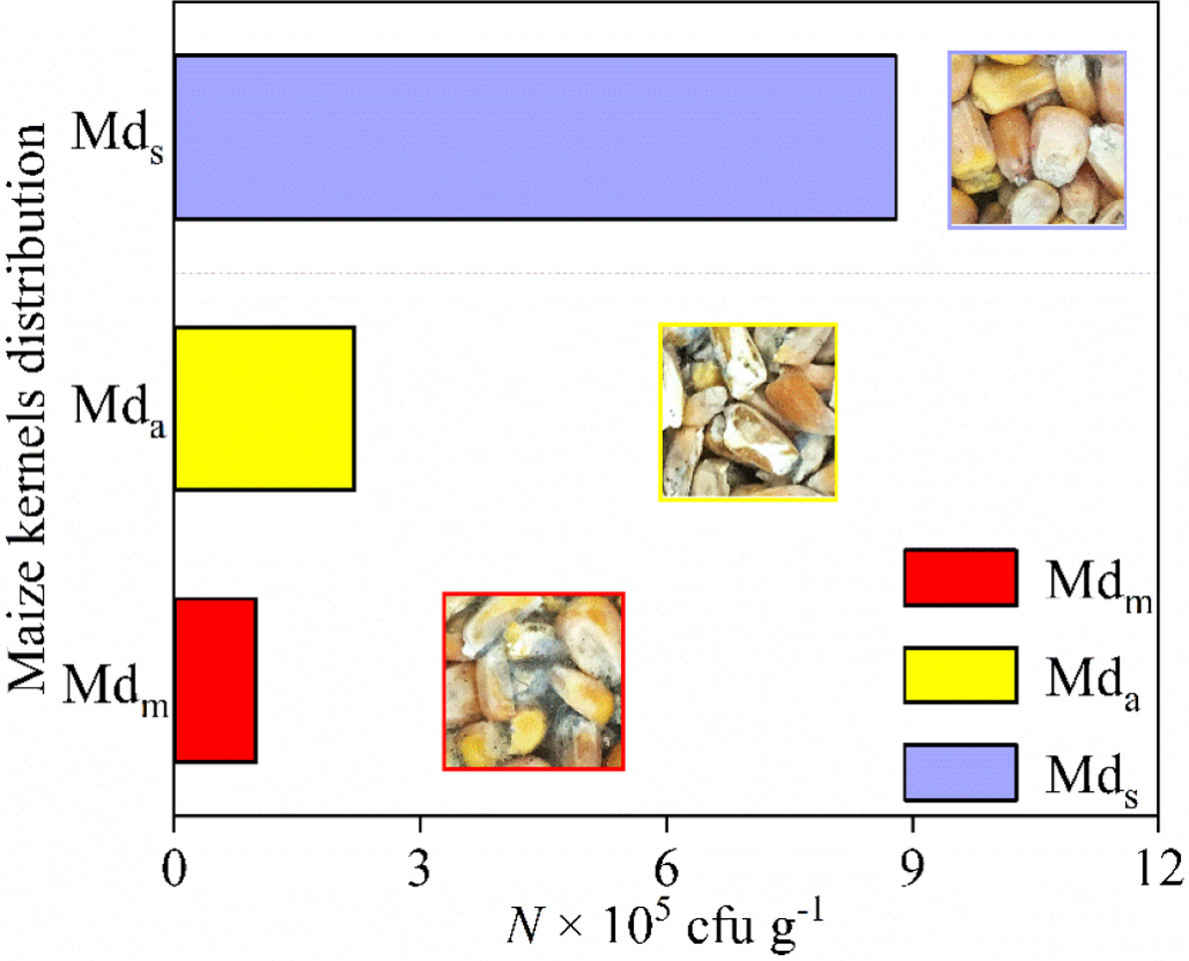
Aerobic plate count of different distribution configurations stored for 150 h

The aerobic plate count of maize kernels is an important parameter to evaluate the bacterial reproductive dynamics and bacterial contamination during storage. The endosperm of maize had celadon hyphae and strong musty off‐odor, and *Penicillium* was the predominant bacterium after 150 h of storage. In addition, the color of less moldy was relatively bright, as shown in Figure 8. The aerobic plate count in the center of the maize sample were 1.0 × 10^5^, 2.2 × 10^5^, and 8.8 × 10^5^ cfu g^−1^ for Md_m_, Md_a_, and Md_s_, respectively. The results show that the segregated grain is more likely to be contaminated as compared with the uniform grain. Similar results were obtained by Navarro and Noyes (2001), who found that the uneven air flow caused by segregation makes the local temperature and relative humidity higher, resulting in the development of fungi and other infestations. Furthermore, this result corresponded very well to the temperature change shown in Figure 7. Fewer molds produced a smaller amount of respiratory heat, with uniform grain.

## 4 HEAT PRODUCTION ANALYSIS

The heat production due to fungi during the test was investigated using a back‐analysis method. First, a finite‐element model was established to simulate the heat variation (i.e., temperature change) caused by physical environmental factors, e.g., room temperature, convection, and moisture content. Second, the heat production induced by biological reason, i.e., fungi, was back‐estimated using the temperature difference (Δ*T*) between tested and simulated results. Finally, a newly reported solution in the biological field by Wu et al. (2020) was employed to theoretically analyze the heat production due to fungi. And the theoretical results and back‐analyzed results were compared with each other.

### 4.1 Numerical modeling of heat transfer

To investigate heat transfer due to physical factors during the testing process, the finite‐element (FE) software COMSOL Multiphysics was used to establish numerical modeling. For simplicity, the maize kernels were assumed as porous media with isotropic properties and in local thermodynamic equilibrium with its surrounding air. Additionally, the laminar air in the void between kernels was incompressible under vertical load. However, the buoyant force and natural convection due to temperature gradient can be considered. The coupled governing equations of heat and moisture transfer of grain bulk include mass equation, energy equation, momentum equation, and moisture transfer equation (Wang et al., 2016). The mass equation can be expressed as:

(3) $$\frac{\partial u_{j}}{\partial x_{j}}=0$$

Where *u*_*j*_ (*j* = 1,2,3) is the air flow rate in the direction *x*_*j*_, *u*_1_ = *u*, *u*_2_ = *u*_3_ = *v*. In a rectangular Cartesian coordinate system, *x*_1_ = *x*, *x*_2_ = *y*, *x*_3_ = *z*. The Brinkman–Darcy formulation was incorporated with the maize domain to represent the airflow. The thermophysical property of air is considered constant, but the buoyant force linearly varies with temperature change. The momentum satisfies the Boussinesiq's approximation, then:

(4) $$\rho_{a}\frac{\partial u_{i}}{\partial t}+\frac{\rho_{a}u_{j}}{\phi }\frac{\partial u_{i}}{\partial x_{j}}=-\frac{\partial p}{\partial x_{i}}+\frac{\partial }{\partial x_{j}}\left[\mu \frac{\partial u_{i}}{\partial x_{j}}\right]+\rho_{0}\mathrm{g\beta}\left(T-T_{0}\right)-\frac{\phi \mu u_{i}}{K}$$

Where *ρ*_a_ is the density of air, *u*_*i*_ is the speed of airflow, *t* is the storage period, *p* is the air pressure, *ф* is the porosity of maize bulk, and *K* is the permeability of maize bulk. *T* is the temperature of the air and maize kernels, *ρ*_0_ is the density of air at the reference temperature *T*_0_, *g* is the gravity vector, *β* is the coefficient of volumetric expansion of the air, and *μ* is the viscosity of air.

As there is a local thermodynamic equilibrium between maize kernels and the surrounding air, the governing conservation equation of thermal energy is as follows:

(5) $$\rho_{b}c_{b}\frac{\partial T}{\partial t}+\left(\rho_{a}c_{a}\right)u_{j}\frac{\partial T}{\partial x_{j}}=\frac{\partial }{\partial x_{j}}\left[k_{b}\frac{\partial T}{\partial x_{j}}\right]+\rho_{b}h_{s}\frac{\partial W}{\partial t}$$

Where *c*_a_ is the specific heat of air; *ρ*_b_, *c*_b_, and *k*_b_ are the dry density, specific heat, and the effective thermal conductivity of maize bulk, respectively; *h*_s_ is the heat sorption of water on maize; and *W* is the moisture content of maize in dry basis. The last term on the right side presents the water absorption and desorption feature of the maize. The moisture content of maize kernels and the absolute moisture content of the air in pores should conserve mass conservation in the process of convection and diffusion. Thus, the moisture balance formula is written as:

(6) $$\frac{\partial }{\partial t}\left[\left({\varepsilon \rho }_{a}w\right)+\left(\rho_{b}W\right)\right]+\rho_{a}u_{j}\frac{\partial w}{\partial x_{j}}=\frac{\partial }{\partial x_{j}}\left[\frac{D_{v}\varepsilon }{\tau }\frac{\partial }{\partial x_{j}}\left(\rho_{a}w\right)\right]$$

Where *τ* is the tortuosity factor of maize bulk, *D*_*v*_ is a dimensionless rate coefficient for moisture exchange between air and maize kernels, and *w* is the moisture content of water vapor in the air on a dry basis.

**FIGURE 9:**
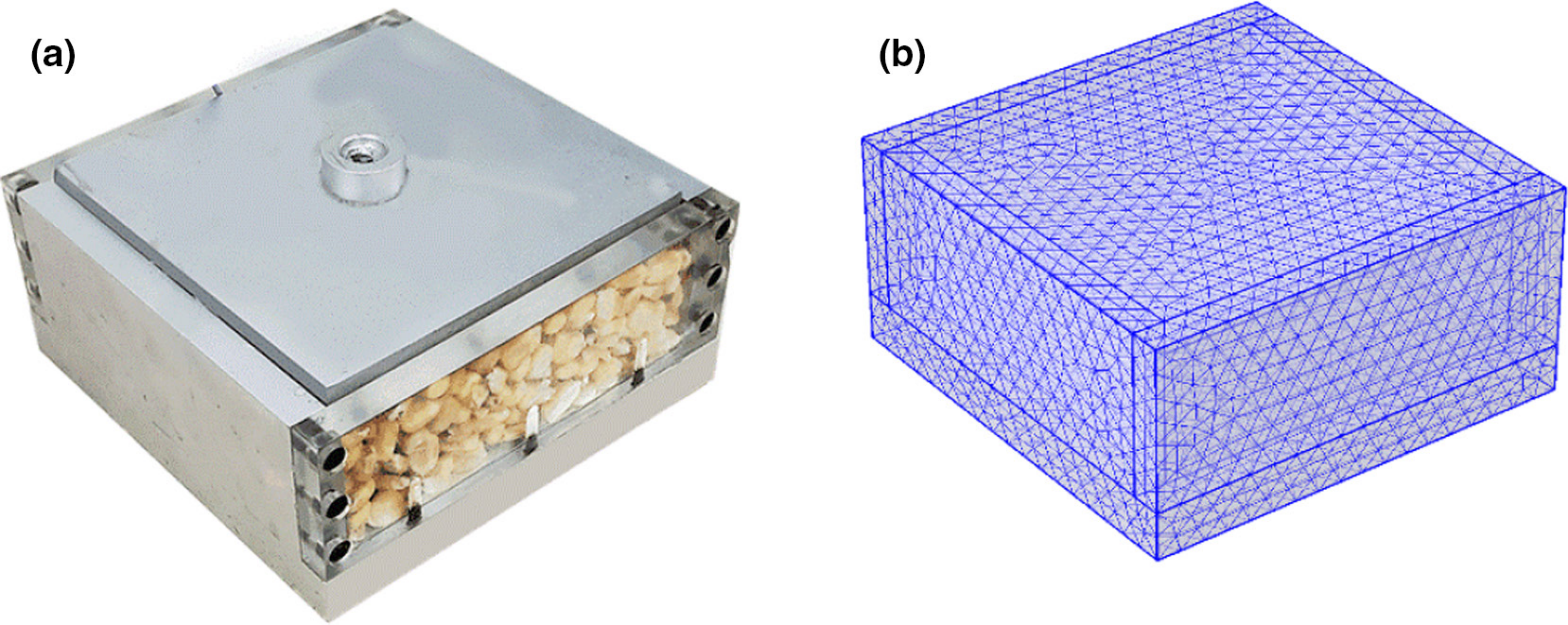
Illustration of test (a) and numerical mesh (b)

To verify the effectiveness of the numerical model, physical heat transfer for maize was investigated with both testing and FE methods. The tests were conducted by placing the sample within the model box (including the top plate) in a freezer. Before testing, the model box, top plate, and spread‐open kernels were placed in the freezer with the door open for hours to reach a uniform initial temperature. Then, the whole maize kernels were put into the box and covered by the top plate. The tests were begun after closing the door and the temperature of the freezer was set at 6°C. The test only lasted for 345 min. Therefore, the heat production by fungi could be ignored. Two T‐type thermocouples were set at the central point of the sample and in the freezer to monitor the temperature change. The material parameters of the model box and maize used in FE analysis are listed in Table 1 and the sources of the parameters are specified as well. The illustration of the test and numerical mesh is shown in Figure 9. It was noted that the maize bulk was basically sealed by the box and top plate during the test. Thus, there is almost no air convection between the pores and the maize moisture changed a little during the test. As a result, the flow rate of air was assumed to be zero and water evaporation was not considered.

**TABLE 1:** The material parameters

Material	Property	Value
Aluminum alloy	Thickness (*l*_A_)	0.01 m
	Thermal conductivity (*k*_A_)	201 W m^−1^ °C^−1^
	Density (*ρ*_A_)	2720 kg m^−3^
	Specific heat (*c*_A_)	90.64 J kg^−1^ °C^−1^
PMMA	Thickness (*l*_P_)	0.01 m
	Thermal conductivity (*k*_P_)	0.19 W m^−1^ °C^−1^
	Density (*ρ*_P_)	1180 kg m^−3^
	Specific heat (*c*_P_)	1424 J kg^−1^ °C^−1^
Air	Air thermal conductivity (*k*_*a*_)	0.025 W m^−1^ °C^−1^
	Air density (*ρ*_a_)	1.205 kg m^−3^
	Air specific heat (*c*_a_)	1006 J kg^−1^ °C^−1^
	Air tortuosity factor (*τ*)	1.2
	Air viscosity (*μ*)	1.79 × 10^−5^ Pa s
	Saturation vapor pressure of water vapor (*p*_sat_)	6 × 10^25^ × T^−5^ × exp (−6800/T) (ASAE, 2017b)
	Vapor pressure of water (*p*_v_)	*p*_sat_ − *p*_sat_·exp[−1.23 × 10^−5^·(*T* + 64.35)·(100·*W*)^2.56^]
	Rate coefficient for moisture exchange between air and maize kernels (*D*_*v*_)	2000exp(−5094/*T*)
Maize	Maize moisture content (*M*_maize_)	9.7 ± 0.03%
	Maize density (*ρ*_maize_)	768 kg m^−3^
	Maize thermal conductivity (*k*_maize_)	0.12 W m^−1^ °C^−1^
	Maize specific heat (*c*_maize_)	2223 J kg^−1^ °C^−1^
	Maize permeability (*K*_maize_)	1.9 × 10^−9^ m^2^

**FIGURE 10:**
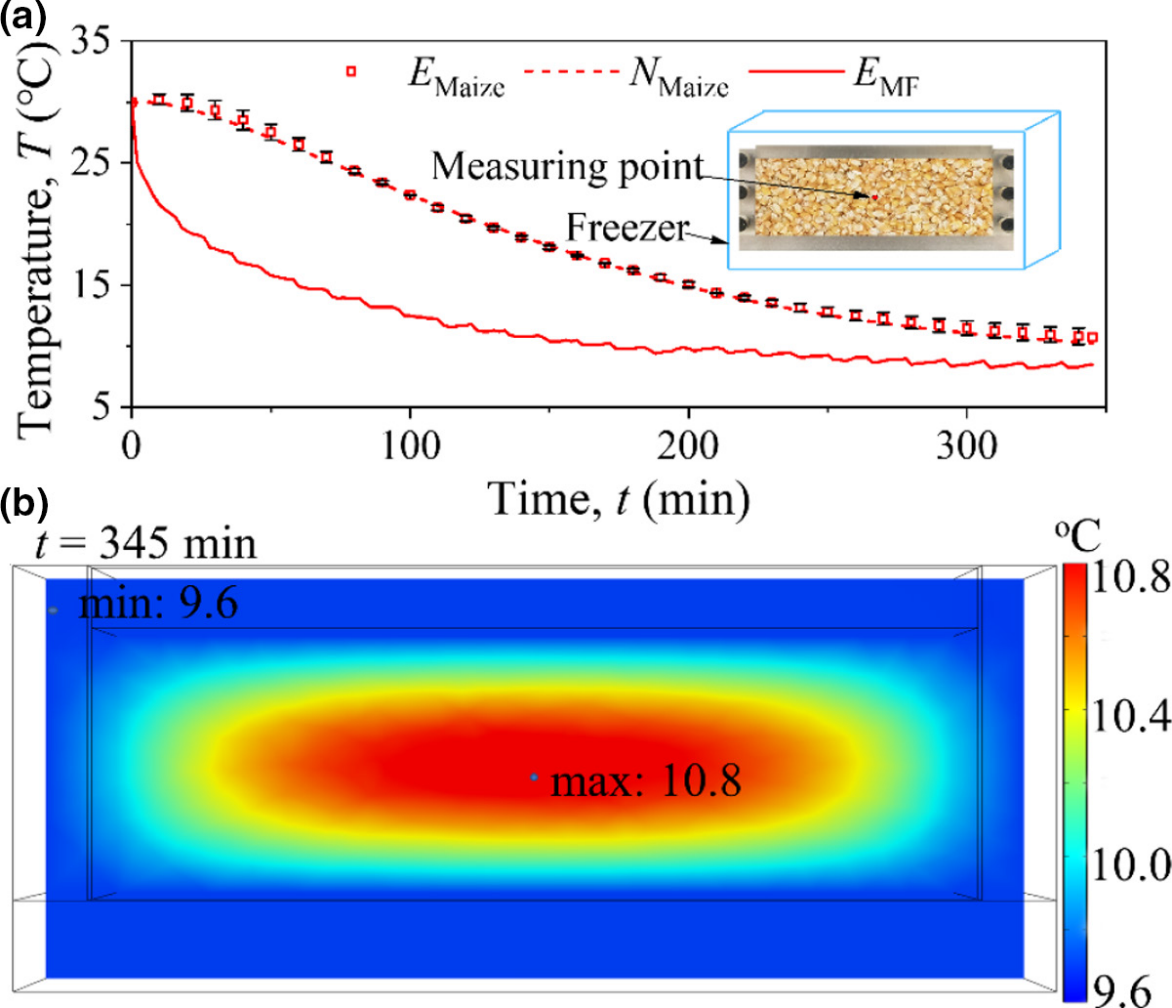
Heat transfer in low‐temperature environment, (a) comparison of experimental and simulation results and (b) temperature cloud charts of mid‐vertical plane

The tested and numerical results of *T* at the central point were compared in Figure 10a. After placing into the freezer, *T* gradually decreased from 30°C around to 6°C. The higher heat of grain bulk is transferred to the box wall under the action of a large temperature difference, and the temperature at the measuring point is the highest at 345 min, that is, 10.8°C, as shown in Figure 10b. The temperature of grain bulk still tends to decrease. The agreements of tested and simulated results are very good. It is worth noting that the tested *T* is slightly higher than the numerical value for maize in the late stage, e.g., after 250 min. The reason is that the biochemical heat production is not zero even under the very low freezer temperature of 6°C. In conclusion, the heat transfer considering various physical factors, such as air convection, water migration and evaporation, and local thermodynamic equilibrium, could be well simulated using the established FE model. This model was used to investigate the physical heat transfer behavior of the maize bulk under vertical pressure.

### 4.2 Numerical results and analysis

**FIGURE 11:**
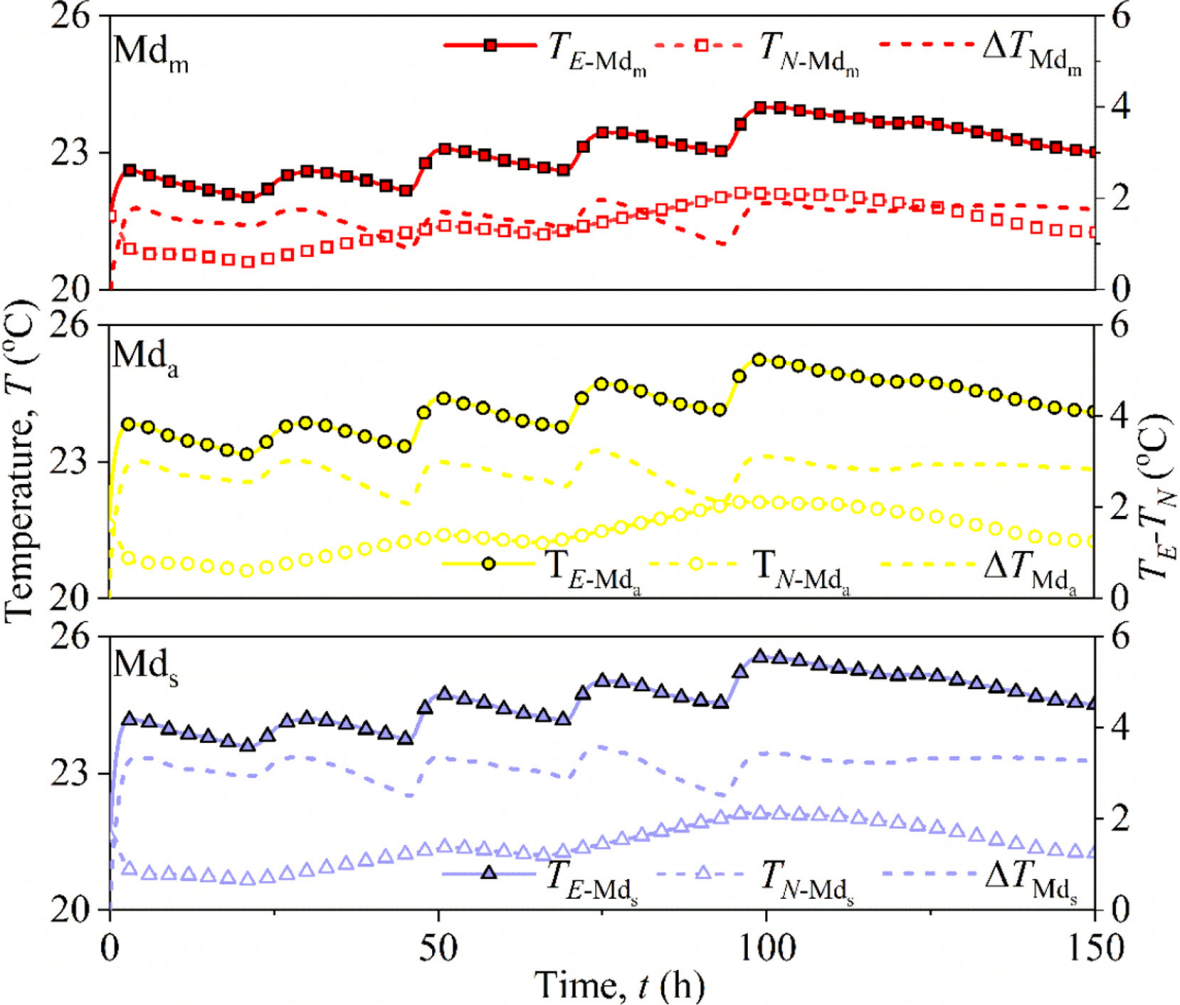
Measured temperature changes fungal heating and temperature changes with no fungus in three configurations of maize kernel distribution, and the corresponding temperature rise due to fungal heating

Using the verified model mentioned in the previous section and considering vertical pressure, the variation in temperature (*T*) at the central point was simulated. The parameters involved in the FE simulation for maize are shown in Table 2. During the simulation, the maize bulk is assumed as a porous medium with isotropic properties, and the vertical pressure‐induced compression (Δ*h*), considered by the input value of density (*ρ*_b_), was calculated using Δ*h* step by step, according to the loading procedure. Similarly, the variation in porosity and thermal conductivity during the test were considered step by step as well. The experimental result and simulated result are designated as *T*_*E*_ and *T*_*N*_, respectively. Figure 11 presents the temperature variations in *T*_*E*_ and *T*_*N*_ for the three groups of maize bulk (Md_m_, Md_a,_ and Md_s_). Clearly, it can be observed that *T*_*E*_ is higher than *T*_*N*_ and the temperature difference Δ*T* (*T*_*E*_ − *T*_*N*_) varies with distribution configuration. Compared with numerical *T*_*N*_ which only includes physical effect, the test *T*_*E*_ resulted from physical heat transfer and biochemical effect. The temperature difference/rise actually is a consequence of heat accumulation due to a biochemical effect. Generally, the segregated grain, e.g., Md_s_, yields larger Δ*T*, and the uniform grain, e.g., Md_m_, yields smaller Δ*T*. The average value of Δ*T* is 1.6°C in Md_m_, and these values were 2.8°C and 3.2°C in Md_a_ and Md_s_, respectively. This result is proportional to the APC in Figure 8, which indicates that mildew is one of the main reasons for temperature rise in grain storage.

**TABLE 2:** The parameters involved in FE simulation for maize

Material property	Value
Moisture content (*M*)	22.9 ± 0.06%
Mass (*m*)	0.4 kg
Initial height (*h*_0_)	0.0414 m (Md_m_)
	0.0413 m (Md_a_)
	0.0432 m (Md_s_)
Maize bulk density (*ρ*_b_)	*m*/0.0144 × (*h*_0_ − Δ*h*)
Particle density (*ρ*_s_)	1234.8 ± 1.5 kg m^−3^
Porosity (*ф*)	1 − *ρ*_b_/*ρ*_s_
Specific heat (*c*_b_)	2223 J kg^−1^ °C^−1^
Thermal conductivity (*k*_b_)	0.0902 + 1.165 × 10^−4^ *ρ*_b_
Heat of sorption of water on maize (*h*_s_)	2476 kJ kg^−1^
Moisture content (dry basis) (*W*)	*M*/(1‐*M*) × 100%
Moisture content of air (*w*)	$\frac{p_{v}}{p_{\mathrm{sat}}}\frac{\left(6800/T-5\right)}{T/p_{\mathrm{sat}}}+p_{s}\left(1.23\times 10^{-5}\cdot W^{2.56}\right)\left(1-\frac{p_{v}}{p_{\mathrm{sat}}}\right)$

Owing to the low thermal conductivity of air between the pores of the kernels, the heat conduction is slow and the heat generated by initial fungi inside the grain bulk cannot be quickly dissipated. Increased temperature in grain bulk further provides a favorable environment for subsequent fungal (Chang, 1986; Suleiman et al., 2018). This cycle of “heat conduction – temperature rise – fungal growth” replies on the initial condition significantly. It is well known that segregation causes non‐uniformity of airflow and decreases the heat transfer efficiency (Navarro & Noyes, 2001). The rate of development of the “heat conduction – temperature rise – fungal growth” cycle is the highest for this case. This result is consistent with the above APC test results.

### 4.3 Estimating the heat produced by fungi

Wu et al. (2020). proposed a framework for estimating the rate of heat production (*Q*) by fungi in grain storage. Note that they regarded the biochemical heat production uniquely as the heat production by fungi. Although the biochemical and fungal effects on heat production are different, the difference can be generally ignored because of the biological effect, i.e., mildew dominates in heat production. In this section, Wu et al.’s solution was used to estimate the heat production by fungi using the test and numerical data mentioned in the previous section (Wu et al., 2020). As indicated by Wu et al. (2020), we know that some of the heat production by fungi (*E*_T_) is used to rise the temperature of the system, some heat is likely lost due to conduction (*E*_C_) and convection (*E*_V_), and some maybe consumed during the water phase change (*E*_E_), e.g., from liquid to vapor. In this study, *E*_C_ was not considered as the sample was nearly sealed and *E*_*E*_ was also considered ignorable because the moisture content only changed a little. Therefore, the heat produced by fungi in the maize bulk can be determined as follows:

(7) $$Q=f\left(\Delta T^{\prime }\right)=E_{T}+E_{C}$$

Where Δ*T′* is the temperature difference in the maize bulk before and after fungus emergence. Heat absorbed by maize and the specific heat capacity of maize bulk with different distribution configurations were calculated using the following formula (ASAE 2017b; Wu et al., 2020):

(8) $$E_{T}=\frac{c_{b}\rho_{b}\Delta V\Delta T^{\prime }}{\Delta V}=c_{b}\rho_{b}\Delta T^{\prime }$$

where ΔV is the spatial range affected by fungal activity, m^3^.

The conduction heat loss (*E*_C_) was determined by considering heat transfer from the geometric center of the maize sample to the wall of the testing box. The calculation space is Δx=0.12m and Δy=0.12m, and Δz is the height of the sample at the corresponding time (ΔV=ΔxΔyΔz). Taking into account the sum of the conduction heat loss on the six sides of the testing box, according to Fourier's law, *E*_*C*_ can be calculated as follows:

(9) $$E_{C}=\frac{k_{b}\Delta t}{\Delta x\Delta y\Delta z}\sum_{i=1}^{6}\frac{A_{i}\Delta T_{i}}{l_{i}}$$

where Δt is the storage time, *A*_*i*_ is the area of one of the six faces of the testing box, and $\Delta T_{i}$ is the temperature difference between the center of the geometric center of the maize sample and the corresponding face *A*_*i*_ measured at a given time *t*. $l_{i}$ is the distance between the center of the geometric center of the maize sample and the corresponding face *A*_*i*_.

Incorporating Equation (8) and Equation (9) into Equation (7), the heat generated by fungal activity during maize storage can be expressed as:

(10) $$Q=E_{T}+E_{C}=c_{b}\rho_{b}\Delta T^{\prime }+\frac{k_{b}\Delta t}{\Delta x\Delta y\Delta z}\sum_{i=1}^{6}\frac{A_{i}\Delta T_{i}}{l_{i}}$$

**FIGURE 12:**
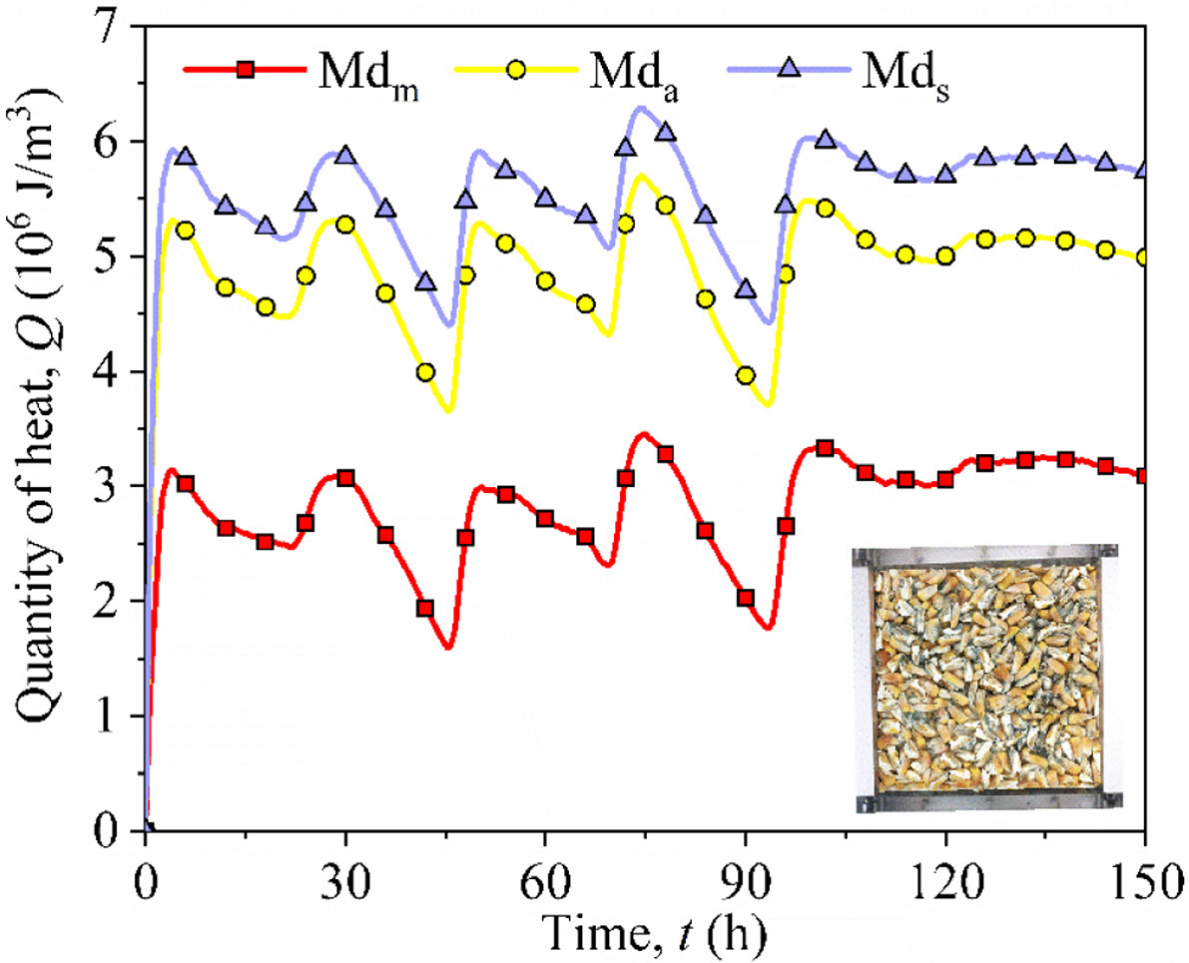
Variation in heat production by fungi in three configurations of maize kernel distribution

In this investigation, the value of temperature difference before and after fungi emergence (Δ*T′*) can be equivalently regarded as the difference between the test and numerical results (Δ*T*). As mentioned above, the reason for Δ*T* between test and simulation is whether the biochemical effect or fungi was considered. The numerical result from COMSOL Multiphysics can only include the physical factors in the simulation. Using the test and numerical temperature difference, heat production (*Q*) by fungi was evaluated according to Wu et al.’s (Wu et al., 2020) solution as shown in Figure 12. The average heat was the least in Md_m_ with 2.8 × 10^6^ J m^−3^, and Md_a_ and Md_s_ were 1.7 and 2 times more than Md_m_. It is clear that this variation in *Q* with time agreed very well with the APC and temperature results (Figures 8 and 11).

## 5 DISCUSSION AND LIMITATION

In this study, the broken kernels inducing different types of segregation were considered and the impact on creep compression and mildew was investigated. Examining the temperature in Figure 7 along with the APC (Figure 8) and the heat production (*Q*) by fungi (Figure 12) reveals the general characteristics of the effects of maize kernels distribution configurations on the mildew in a silo. When a maize mixture was loaded into a silo, three representative segregation configurations were observed, i.e., mixing, alternating distribution, and segregation. Different distribution configurations resulted in different compression deformation and porosity distribution. This led to the difference in heat transfer efficiency in locations in the grain bulk and further resulted in serious grain problems. In a complete separation state, the larger pores among the whole kernels on the upper layer provide relatively sufficient oxygen for the development of fungi in the center of the sample. In the initial storage stage, the fast‐growing fungi released a lot of heat and raised the grain temperature, as seen in Figure 7b. The contact area among the kernels is relatively small and the pores are relatively large. The low heat transfer efficiency caused by these reasons makes heat accumulate and further stimulates the growth of fungi. The growth of fungi produces heat and water through respiration, causing the temperature and moisture to rise, which in turn will accelerate the fungal growth. The development of this cycle of “heat conduction ‐ temperature rise ‐ fungal growth” is easy to cause undesired problems of grain storage. Therefore, necessary measures should be taken to reduce segregation in the loading process of a grain silo.

Although this study found the effect of segregation on mildew, the segregation mechanism of storage grain bulk is complex. The broken kernel shape is not only whole kernel and half kernel, but also the distribution form is not limited to these three regular configurations. The mildew of grain in the silo is the result of the comprehensive action of temperature, moisture content, vertical pressure, segregation, and other factors, and the study of the effects of such comprehensive factors on grain mildew in a large‐scale container is recommended.

## 6 CONCLUSION

In this study, the compression of three configurations of maize kernels distribution (Md_m_, Md_a,_ and Md_s_) under 200 kPa was investigated followed by aerobic plate count, and then the heat production by fungi was studied by numerical simulation and analytical research. From this study, the main conclusions are as follows:

1. The maize bulk deforms greatly under vertical load, and the compression increased with the increase in the degree of separation. The deformation in the stage of load increase was the main cause of compression deformation, which accounted for 80.6%, 81.3%, and 81.8% of the total deformation of Md_m_, Md_a_, and Md_s_ respectively, and the porosity in this stage decreases by 22.2%, 22.7%, and 24.3%, respectively. The three‐element Schiffman model can represent the creep behavior of different distribution configurations.
2. The average temperature and the aerobic plate count of maize increased with the increase in the degree of separation. The average temperature of Md_m_, Md_a_, and Md_s_ were 7.53%, 12.98%, and 14.76% higher than the average room temperature, respectively. The aerobic plate count of Md_m_, Md_a_, and Md_s_ was 1.0 × 10^5^, 2.2 × 10^5,^ and 8.8 × 10^5^ cfu g^−1^ stored for 150 h, respectively.
3. A finite‐element model was for accurately simulating the heat variation caused by physical environmental factors. The heat production by maize bulk fungi was quantified using the test and numerical temperature difference. The average heat was the least in Md_m_ with 2.8 × 10^6^ J m^−3^, and Md_a_ and Md_s_ were 1.7 and 2 times more than Md_m_. The heat was related to the segregation configurations and agreed very well with the APC and temperature results.

## Data Availability

The test data was has been presented in the paper, and other data used to support the findings of this study are available from the corresponding author upon request.
